# Estimating Visual Field Mean Deviation using Optical Coherence Tomographic Nerve Fiber Layer Measurements in Glaucoma Patients

**DOI:** 10.1038/s41598-019-54792-w

**Published:** 2019-12-06

**Authors:** Ou Tan, David S. Greenfield, Brian A. Francis, Rohit Varma, Joel S. Schuman, David Huang

**Affiliations:** 1 Casey Eye Institute, Oregon Health & Science University, Portland, USA; 2 Bascom Palmer Eye Institute, University of Miami, Coral Gables, USA; 3 Doheny Eye Institute, David Geffen School of Medicine at UCLA, Los Angeles, USA; 4 Southern California Eyecare and Vision research Institute, Los Angeles, USA; 5 New York University Langone Medical Center, New York, USA

**Keywords:** Prognostic markers, Translational research

## Abstract

To construct an optical coherence tomography (OCT) nerve fiber layer (NFL) parameter that has maximal correlation and agreement with visual field (VF) mean deviation (MD). The NFL_MD parameter in dB scale was calculated from the peripapillary NFL thickness profile nonlinear transformation and VF area-weighted averaging. From the Advanced Imaging for Glaucoma study, 245 normal, 420 pre-perimetric glaucoma (PPG), and 289 perimetric glaucoma (PG) eyes were selected. NFL_MD had significantly higher correlation (Pearson R: 0.68 vs 0.55, p < 0.001) with VF_MD than the overall NFL thickness. NFL_MD also had significantly higher sensitivity in detecting PPG (0.14 vs 0.08) and PG (0.60 vs 0.43) at the 99% specificity level. NFL_MD had better reproducibility than VF_MD (0.35 vs 0.69 dB, p < 0.001). The differences between NFL_MD and VF_MD were −0.34 ± 1.71 dB, −0.01 ± 2.08 dB and 3.54 ± 3.18 dB and 7.17 ± 2.68 dB for PPG, early PG, moderate PG, and severe PG subgroups, respectively. In summary, OCT-based NFL_MD has better correlation with VF_MD and greater diagnostic sensitivity than the average NFL thickness. It has better reproducibility than VF_MD, which may be advantageous in detecting progression. It agrees well with VF_MD in early glaucoma but underestimates damage in moderate~advanced stages.

## Introduction

Glaucoma is a leading cause of blindness^1,2^, and effective glaucoma management requires early detection, followed by careful evaluation and monitoring to identify those at the highest risk for disease progression and vision loss. This allows the rational use of medical, laser, and surgical treatments, all of which have significant cost, compliance, and safety issues. Visual field (VF) test is the current standard to monitor glaucoma progression. However, VF testing is subjective, time-consuming, and poorly reproducible. Quantitative imaging of the optic nerve head (ONH) and retina with optical coherence tomography (OCT)^3^ are widely used in diagnosis and monitoring of glaucoma^4,5^. But the overall peripapillary nerve fiber layer (NFL) thickness correlates poorly with VF mean deviation (MD)^6,7^. Furthermore, the speed of glaucoma progression as measured by OCT, such as NFL and macular ganglion cell complex (GCC) thinning in µm/year poorly correlates with the rate of VF changes as measured in MD trend in dB/year or Visual Field Index (VFI) trend in %/year ^8–12^. Thus it is difficult to clinically judge whether glaucoma is progressing rapidly or not based on OCT structural measurements.

A major reason for the frequent discordance between OCT and VF results is the way in which they are scaled. OCT measures NFL and GCC in µm units, which is on a linear scale. VF maps and parameters are measured in decibel (dB) units on a logarithmic scale. Differences also exist in the strategy to provide summary data for OCT and VF testing. For example, the NFL thickness is weighted by the length along a peripapillary circle. In contrast, VF_MD is weighted by the VF area.

In this study, we hypothesized that reducing the differences in scaling and weighting could improve the correlation between VF and OCT measurements. We developed a method to estimate the VF_MD using the circumpapillary NFL thickness profile measured by OCT in the same eye. The method converts NFL thickness to a dB scale and averages it using VF area weighting. We then assessed whether the resulting NFL_MD has advantages over the commonly used overall NFL thickness in terms of diagnostic accuracy, staging accuracy, and correlation with VF_MD. Finally, the potential for more sensitive progression detection is evaluated by looking at between-visit retest variability.

## Results

### Characteristics of the study participants

Two hundreds and forty five normal eyes from 124 participants, 420 PPG eyes from 245 participants, and 289 PG eyes from 192 participants in the AIGS dataset had acceptable-quality OCT and VF data. Eyes in both the PPG and PG groups had significantly older age, longer axial length, higher IOP and, thinner RNFL than the normal group (Table 1). In addition, eyes in PG group also had thinner central cornea, worse visual field MD and PSD than the normal. Although the age differences were statistically significant, they were small (2–3 years). In the PG group, 213 eyes had early PG (MD > −6 dB, stage 1), 47 eyes had moderate PG (MD between −6 and −12 dB, stage 2), 29 eyes had severe PG (MD < −12 dB, stage 3) according to the modified Hodapp-Parrish-Anderson (HPA) staging criteria^7^. The PPG eyes had HPA stage 0, as their PSD and GHT values were normal by definition.

**Table 1:** Characteristics of the Study Population.

Characteristics	Normal (N)	Pre-perimetric Glaucoma (PPG)	p-value N v. PPG	Perimetric Glaucoma (PG)	p-value N v. PG
Number of participants (eyes)	124 (245)	245 (420)	N/A	192 (289)	N/A
Age (years)	58.4 ± 9.3	61.0 ± 9.5	0.001	63.0 ± 9.6	<0.001
Female (%)	64.2	60.9	0.388	61.6	0.537
African Decent (%)	6.5	12.9	0.009	10.4	0.110
Axial length (mm)	23.7 ± 1.0	24.3 ± 1.3	<0.001	24.3 ± 1.3	<0.001
Corneal thickness (µm)	562 ± 33	558 ± 37	0.180	545 ± 37	<0.001
IOP (mmHg)	14.9 ± 2.2	16.2 ± 2.8	0.010	17.3 ± 3.3	<0.001
VF MD (dB)	−0.0 ± 1.1	−0.5 ± 1.1	0.055	−4.7 ± 4.4	<0.001
VF PSD (dB)	1.5 ± 0.3	1.8 ± 0.5	0.250	5.7 ± 4.1	<0.001
Overall NFLT (µm)	100.5 ± 8.4	96.0 ± 10.0	<0.001	84.2 ± 11.3	<0.001

The characteristics of the study participants were averaged over the 4 consecutive study visits except for axial length and central corneal thickness, which were only measured at baseline. IOP = intraocular pressure; VF = visual field; MD = mean deviation; PSD = pattern standard deviation; NFLT = nerve fiber layer thickness.

Though African decent is significantly more in PPG group than normal group. No adjustment was applied because no significant difference was found between African decents and European decents in AIGS normal group for NFL parameters.

### Normal reference and floor values

The normative reference values for sector and overall NFL thickness were calculated from 245 normal participants with age and axial length correction (Table 2).

**Table 2:** Intercept and slope for overall and sectoral NFL thickness estimation.

		Y-Intercept (µm)	Age (µm/year)	Axial Length (µm/mm)
Overall NFL Thickness		188.0	−0.14	−3.38
Inferior quadrant NFL thickness		246.3	−0.10	−4.93
Sectoral NFL Thickness	TU1	124.1	−0.14	−2.06
	TU2	141.1	−0.13	−1.64
	ST2	199.4	−0.26	−2.30
	ST1	225.9	−0.12	−3.54
	SN1	187.5	−0.08	−3.06
	SN2	202.5	−0.29	−3.31
	NU2	235.9	−0.32	−5.19
	NU1	203.5	−0.17	−5.00
	NL1	151.6	−0.07	−3.39
	NL2	189.1	−0.13	−4.22
	IN2	241.2	−0.19	−5.40
	IN1	332.9	−0.07	−8.71
	IT1	290.0	−0.05	−5.98
	IT2	121.9	−0.10	0.36
	TL2	72.5	−0.15	0.51
	TL1	89.0	−0.03	−1.18

The floor value as a percentage of the reference NFL thickness was found to be 45% by pooling all sectors. The simplifying assumption that the floor percentage is the same for all sectors was necessary as the worst sectors were always found to be inferotemporal or superotemporal. The floor percentage was similar among the inferotemporal and superotemporal sectors with no clear pattern of difference.

### Agreement between NFL and VF parameters

**Figure 1:**
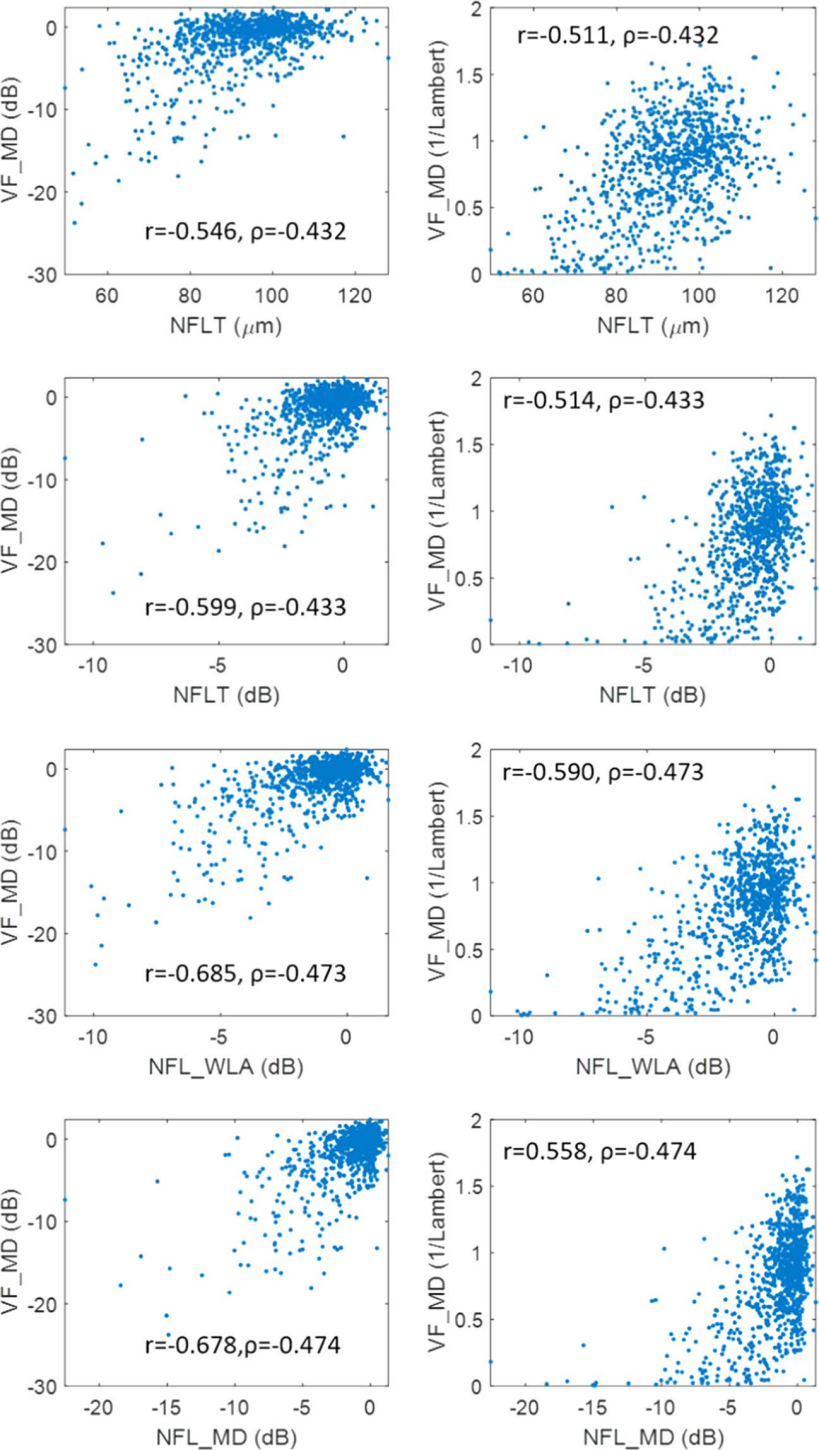
Correlation of optical coherence tomography retinal nerve fiber layer (NFL) parameters and visual field mean deviation (VF_MD). Abbreviations: r - Pearson correlation coefficient; ρ - Spearman’s rank correlation coefficient; WLA – weighted logarithmic average; NFLT-NFL thickness;

The overall average NFL thickness in µm had fair correlation with VF_MD, but the relationship was highly nonlinear (Fig. 1). This was improved by simply converting from µm to dB scale (Fig. 1). Altering the NFL averaging procedure to use dB scale and VF area weighting yielded NFL_WLA_, which had even better correlation with VF_MD (Fig. 1). Removing the residual nonlinearity yielded NFL_MD, which had the best correlation with VF_MD (Fig. 1). In the five-fold cross validation used to evaluate NFL_MD performance, the quadratic formulas is slightly different for each fold. The quadratic formula based on the fitting of all participants is:

$${\rm{NFL}}\_{\rm{MD}}=0.864\,\ast \,({{\rm{NFL}}}_{{\rm{WLA}}})-0.075\,\ast \,{({{\rm{NFL}}}_{{\rm{WLA}}})}^{2}$$

The correlation between NFL_MD and VF_MD (dB) was significantly (p < 0.014) higher than that between overall average NFL thickness average (either in µm or dB scale) and VF_MD, for both Pearson and Spearman coefficients (Fig. 1). If the VF_MD (dB) was transformed into VF sensitivity (1/Lambert), the correlation with NFL thickness actually became worse.

**Figure 2:**
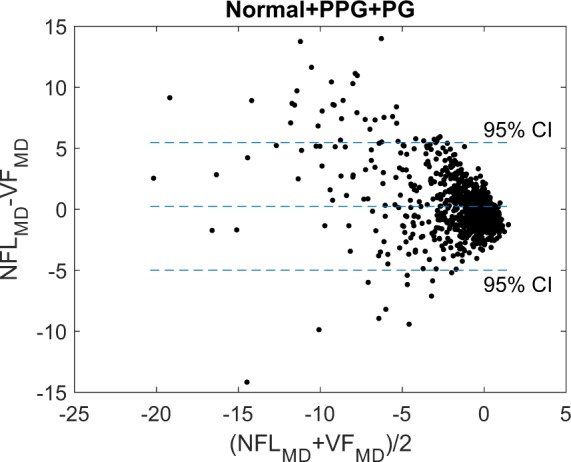
Bland-Altman analysis of the agreement between nerve fiber layer-mean deviation (NFL_MD) and visual field-mean deviation (VF_MD). Data from normal, pre-perimetric glaucoma, and perimetric glaucoma groups are combined.

**Figure 3:**
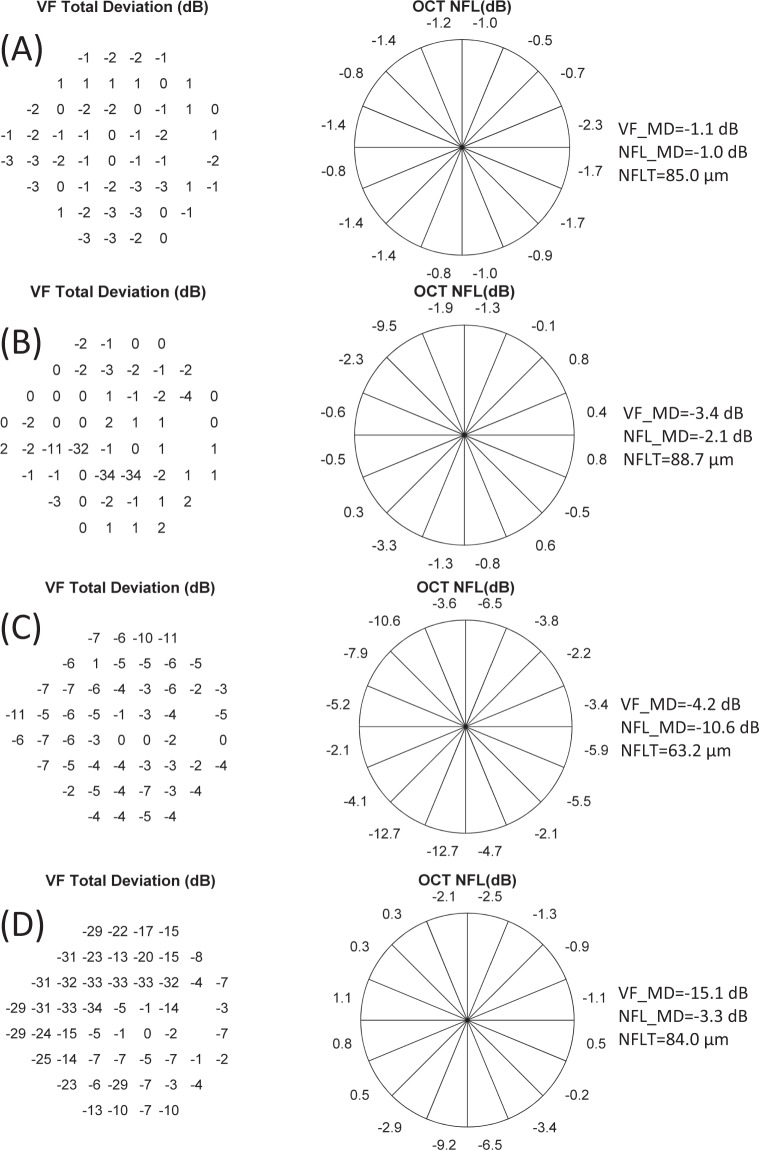
Examples showing how nerve fiber layer-mean deviation (NFL_MD) could behave differently from visual field mean deviation (VF_MD) and overall nerve fiber layer thickness (NFLT) as diagnostic parameters. Visual field (VF) total deviation maps are shown in the left column. The sectoral retinal nerve fiber layer (NFL) thickness in decibel (dB) scale is shown in the middle column. (**A**) a normal eye with diffusely thin NFL; (**B**) an early perimetric glaucoma (PG) eye with focal VF and NFL defects; (**C**) an early PG eye with NFL_MD was more than 6 dB worse than VF_MD; (4) an advanced PG eye with NFL_MD more than 11 dB better than VF_MD.

Difference analysis (Table 3) and Bland-Altman analysis (Fig. 2) showed that the agreement between NFL_MD and VF_MD was good in PPG group, fair in the early PG group, and poor in the moderate PG group and advance-to-severe PG group. There was an average bias toward better NFL_MD than VF_MD in the moderate to severe PG groups. The standard deviation of the difference between NFL_MD and VF_MD increased with increasing glaucoma severity. There were several outliers in the PPG, early PG, and moderate PG groups that had much worse NFL_MD than VF_MD (Figs. 2, 3). Whereas the NFL_MD was generally better than the VF_MD in the advanced-to-severe PG group. Overall, NFL_MD agreed well with VF_MD in PPG and early PG stages. But in the later stages of glaucoma (moderate to severe PG), NFL_MD tend to underestimate glaucoma severity, in comparison to VF_MD.

**Table 3:** Mean Deviations, Cataract Density, and Visual Acuity Stratified by Glaucoma Severity.

Parameter	Normal	PPG	Early PG	Moderate PG	Severe PG
VF_MD (dB)	−0.04 ± 0.79	−0.51 ± 1.15	−2.48 ± 1.17	−8.55 ± 0.00	−15.18 ± 1.65
NFL_MD (dB)	−0.17 ± 0.55	−0.85 ± 1.22	−2.47 ± 1.87	−5.00 ± 3.22	−8.11 ± 4.25
NFL_MD – VF_MD (dB)	−0.13 ± 0.88	−0.34 ± 1.71	0.01 ± 2.08	3.54 ± 3.18	7.17 ± 2.68
Cataract (0–4)	0.59 ± 0.51	0.79 ± 0.55	0.89 ± 0.57	0.79 ± 0.36	0.81 ± 0.50
BCVA (LogMAR)	−0.03 ± 0.07	0.01 ± 0.07	0.02 ± 0.06	0.00 ± 0.08	0.06 ± 0.07

Group mean ± standard deviation. The best-corrected visual acuity (BCVA) was analyzed in the form of the logarithm of minimum angle of resolution (logMAR). LogMAR values of 0, 0.1, and 0.2 are equivalent to Snellen acuity of 20/20, 20/25, and 20/32.

One possible explanation for the discrepancy between NFL_MD and VF_MD in the moderate-to-severe PG stages is cataract severity. Therefore, we examined cataract severity and BCVA in the different stages of glaucoma (Table 3). No significant difference between stages was found.

The agreement between NFL_MD and VF_MD staging of glaucoma severity was compared using the modified Hodapp-Parrish-Anderson classification (Table 4). The NFL_MD staging is based on the value of NFL_MD only: Stage 0–1, NFL_MD > = −6dB; stage 3, NFL_MD < −6dB. The F1 score was used to assess agreement. The F1 score was a better metric than kappa as a metric for agreement in this case because of the imbalance in the chi-square tables (Table 4). The classification agreement was excellent in the PPG group (F1 score 0.99) and good in the PG group (F1 score 0.87). In the PG group, there was a tendency for NFL_MD to under-estimate glaucoma severity stage, compared to VF_MD.

**Table 4:** Staging Agreement between Nerve Fiber Layer and Visual Field Mean Deviations.

		NFL_MD			
		PPG eyes		PG eyes	
		Stage 0–1	Stage 2–3	Stage 0–1	Stage 2–3
VF_MD	Stage 0–1	417	3	201	14
	Stage 2–3	0	0	45	29

The modified Hodapp-Parrish-Anderson glaucoma staging system was used. Abbreviations: NFL – nerve fiber layer; MD – mean deviation; PPG – pre-perimetric glaucoma; PG – perimetric glaucoma; VF – visual field.

In aggregate analysis of all groups, NFL_MD had similarly excellent intra-class correlation as overall NFL thickness for both within-visit repeatability (0.988 vs 0.988) and between-visit reproducibility (0.978 vs 0.968).

The reproducibilities of NFL_MD and VF_MD were also assessed by the pooled root-mean-square residual of linear regression over 4 consecutive visits in glaucoma eyes (Table 5). This could be viewed as the standard deviation between visits adjusted for the glaucoma progression trend between visits. Overall, NFL_MD has better reproducibility than VF_MD (0.35 vs 0.69 dB, p < 0.001). For both NFL_MD and VF_MD, the reproducibility was best at the earliest stage of glaucoma and worsened in the more severe stages. NFL_MD had better reproducibility than VF_MD at all stages and the difference is significantly in PPG and early PG stages.

**Table 5:** Reproducibility of Mean Deviation for Visual Field & Optical Coherence Tomography.

Parameter	PPG	Early PG	Moderate PG	Severe PG
VF_MD (dB)	0.62	0.70	0.84	1.18
NFL_MD (dB)	0.23	0.42	0.68	0.45
p-value	<0.001	<0.001	0.22	0.003

The reproducibility of visual field mean deviation (VF_MD) and OCT-based retina nerve fiber layer-mean deviation (NFL_MD) were estimated by the root-mean-square residual of linear regression from 4 consecutive visits.

The diagnostic accuracy of NFL_MD was compared with VF_MD and the two best NFL diagnostic parameters on linear micron scales (Table 6). For the discrimination between PPG and normal groups, NFL_MD had significantly (p < 0.001) better diagnostic accuracy, as measured by AROC, than overall NFL thickness. NFL_MD also had both higher diagnostic sensitivity at the 95% and 99% specificity cutoff (p < = 0.001, McNemar test) than overall NFL thickness, and inferior NFL thickness (p < = 0.01). For discrimination between the PG and normal groups, NFL_MD had significantly (p < 0.001) higher AROC than overall NFL thickness, marginally higher (p = 0.09) than inferior NFL thickness, and significantly (p < 0.006) higher sensitivity than both micron-scale NFL parameters at both 95% and 99% specificity. Other differences between NFL_MD and other parameters were not significant. Overall, the consistent pattern was that NFL_MD had better diagnostic accuracy than micron scale NFL parameters.

**Table 6:** Diagnostic Accuracy of Nerve Fiber Layer-Mean Deviation Compared to Other Optical Coherence Tomography and Visual Field Parameters.

Discrimination Task	Parameter	AROC	Sensitivity		Cutoff	
		Mean ± SD	95% Specificity	99% Specificity	95% Specificity	99% Specificity
Pre-Perimetric Glaucoma v. Normal	VF_MD	0.571 ± 0.024*	0.141*	0.057*	−2.00	−3.32
	Overall NFLT	0.626 ± 0.023*	0.173*	0.077*	87.7	82.3
	Inferior NFLT	0.648 ± 0.023	0.205*	0.089*	106.1	98.1
	NFL_MD	0.655 ± 0.023	0.240	0.136	−1.23	−1.78
Perimetric Glaucoma v. Normal	VF_MD	0.917 ± 0.013	0.646	0.461*	−2.00	−3.32
	Overall NFLT	0.850 ± 0.019*	0.563*	0.433*	87.7	82.3
	Inferior NFLT	0.879 ± 0.018	0.643*	0.534*	106.1	98.1
	NFL_MD	0.896 ± 0.016	0.702	0.597	−1.23	−1.78

Area under receiver operating characteristic curve (AROC) for visual field mean deviation (VF_MD) and OCT-based retinal nerve fiber layer (NFL) parameters. The NFL parameters are overall average NFL thickness (overall NFLT), inferior quadrant NFLT (Inferior NFLT) and NFL mean deviation (NFL_MD).

*P-value < 0.05 comparing to NFL_MD

- diagnostic accuracy of VF-MD is not calculated because VF is in the selection criteria of pre perimetric glaucoma.

We provided the diagnostic accuracy measures for VF_MD as background information. However, because abnormal VF is an inclusion criterion for the PG group and exclusion criterion for the PPG group, the selection bias makes it difficult to draw conclusions regarding the diagnostic accuracy of VF_MD. But it is remarkable that despite the selection bias in favor of VF_MD, NFL_MD actually achieved a higher diagnostic accuracy.

Several examples are shown to give insight on why NFL_MD might perform differently from overall NFL thickness (micrometer scale) and VF_MD (Fig. 3). The example in Fig. 3A shows that overall NFL thickness could be abnormally low in a normal eye with uniformly thin NFL, but yet NFL_MD could remain within normal limits. This demonstrates how NFL_MD could have improved diagnostic specificity over NFL thickness in people with normally thin NFL. In Fig. 3B, NFL_MD was abnormal due to focal defects in the superotemporal and inferotemporal sectors while the overall NFL thickness remained within normal range because other sectors had above normal thickness (positive sector dB values). This demonstrates how NFL_MD could have improved diagnostic sensitivity because the logarithmic (dB) scale and VF area weighting emphasized focal thinning in the characteristic glaucoma pattern. Figure 3C shows an early PG eye where NFL_MD was much worse than VF_MD, probably because the eye already started with thin NFL prior to glaucoma damage – the pattern of NFL thinning was both diffuse and focal. Figure 3D shows an advanced PG eye where the NFL_MD was much better than VF_MD, probably because the eye started with thicker than average NFL – in sectors less affected by glaucoma the NFL thickness remained above average (positive dB values).

## Discussion

Visual field and OCT measurements are both commonly used for the diagnosis and monitoring of glaucoma^13–15^. Unfortunately, VF parameters and OCT-based NFL thickness parameters do not correlate well with each other^16–18^. This poses challenges in the staging and monitoring of glaucoma, given the potential for discordant functional and structural results.

One reason for the low correlation between NFL and VF is the disparate scales on which they are measured. NFL thickness parameters (i.e. overall, quadrant, octant, and sector averages) are measured using a linear µm scale, while VF parameters (i.e. mean deviation, pattern standard deviation, and visual field index) are measured in dB using a logarithmic scale. To harmonize the two types of measurements, Malik *et al*. suggested that the correlation between VF and NFL should be either in linear to linear scale or logarithm–logarithm scale^19^.

To convert OCT measurements to a scale more consistent with VF testing, investigators have used quadratic, broken stick and logarithmic transformations^16,17,20,21^. Machine learning has also been used to transform OCT information into estimates of retinal sensitivity (a VF measure)^22,23^. In Kihara’s deep learning model, localized slices from B-scans was directly used to estimate the retinal sensitivity point-by-point using a convolutional neural network with a regression output^23^.

Other investigators have converted VF results to a linear scale. Hood *et al*. suggested a linear model to relate NFL thickness and VF sector retinal sensitivity (linear 1/Lambert unit)^24^, using a modified Garway-Heath sector scheme^25^. Hood also showed that it is necessary to subtract the NFL thickness floor value in order to find the best correspondence with linearized VF measures. Wu *et al*. used the similar model on a different structure-function correspondence map generate by Kanamori *et al*.^21^.

We believe that converting OCT measurements to a logarithmic scale is a superior strategy for determining the rate of disease progression, as compared to converting VF parameters to a linear scale. Caprioli *et al*. showed that the worsening of VF_MD, on the usual dB scale, decelerates with respect to time in the more advanced stages^26^. If VF_MD is transformed from dB to linear scale, this nonlinearity would be even more exaggerated, with rapid progression in the early stages and very little change in the later stages. Indeed this is what is found when glaucoma is monitored with OCT NFL measurements on a linear micron scale – there is more rapid progression in early stages and almost no change in the advanced stage^8^. It makes sense that in advanced stages of glaucoma, when there few retinal nerve fibers remain, there would be very little further thinning of the NFL. Yet it is important to monitor the rate of thinning as a percentage of what remains, as even a few μm of thinning at the advanced stages could have large impact on vision and quality of life. Thus, using a logarithmic (dB) scale to measure glaucoma may facilitate change detection across the entire spectrum of glaucomatous disease severity. Statistical considerations also favor the logarithmic scale, as we have found the log-log correlation to be better than linear-linear correlation between VF and NFL thickness (Fig. 1).

In order to improve the correlation with VF_MD, it is insufficient to simply transform the overall NFL thickness from a µm to dB scale. It is necessary to perform the logarithmic transformation on a point or sector basis, and then perform the averaging operation using weights that are proportional to VF area. We demonstrated that this NFL weighted logarithmic average, compared to a simple logarithmic transform of the NFL average thickness, was better correlated with VF_MD. This result is consistent with the finding by some investigators that the correlation between VF and NFL is higher for sectors averages than overall average^18,27^.

The NFL-weighted logarithmic average still exhibited a floor effect in eyes with moderate-to-severe glaucoma. Thus a final quadratic fit was used to obtain the NFL_MD, an OCT-based optimized estimate for VF_MD. Compared to overall NFL thickness using a linear scale, NFL_MD demonstrated much better correlation with VF_MD. The agreement between NFL_MD and VF_MD are good in the PPG and early PG stages, however, NFL_MD still significantly underestimated VF damage in the moderate PG stage and markedly under-estimated VF damage in the advanced-to-severe stages. Thus the clinician needs to exercise caution in applying NFL_MD to glaucoma staging.

There are several reasons for the this discrepancy. The lower limit of −12.8 dB we placed on sector NFL value, is not nearly as low as the worst VF total deviation on a pointwise basis, which has a bottom limit of −33 dB on the Humphrey Field Analyzer^28^. While we could lower the bottom limit to extend the dynamic range of NFL_MD, this would significantly worsen the repeatability from NFL measurement noise. Since our primary goal for developing the NFL_MD was to improve glaucoma monitoring, we want to maintain the reproducibility of NFL_MD over VF_MD across all stages of glaucoma. Thus some remaining discrepancy in the advanced stages of glaucoma may be unavoidable. Other reasons for discrepancy between NFL_MD and VF_MD include cataract, other media opacities and optical aberrations, dry eye, and psychophysical limitations on the subject’s test taking ability. These may explain some outlier points where VF_MD was poor while NFL_MD was near normal. In these cases, NFL_MD may provide a more accurate assessment of glaucoma severity than VF_MD. On the other hand, error in NFL_MD could be introduced by image processing (i.e. segmentation) error and anatomic changes such as retinal edema and epiretinal membrane.

The largest source of discrepancy may be unavoidable variation in NFL thickness within the normal population. The standard deviation of overall NFL thickness in our sample was 8.4 µm, 8.5% of the normal average value of 99.7 µm. Thus 95% confidence interval of NFL_MD would be −1.5 to + 0.9 dB simply from normal population variation. If the eye were to have −6 dB (75%) loss of nerve fibers from baseline, the 95% confidence interval due to the variation from their starting point would be −14.1 to −5.0 dB according to our NFL_MD formula. Thus one can see that the agreement between NFL_MD and VF_MD would deteriorate in the more advanced stages of glaucoma simply due to the variation in normal NFL thickness and its floor value. Although we have reduced this variation by adjusting for age and axial length^29^, most of this variation is random and cannot be adjusted for. Thus the use of NFL_MD in the staging of glaucoma would always be hampered by the fact that each of us is born with a different NFL thickness.

Compared to conventional µm-scale NFL thickness, NFL_MD correlates better with VF_MD. But this correlation is still not good in moderate and severe glaucoma stages, and this poses a limitation for the monitoring of glaucoma progression. For the objective monitoring of glaucoma progression in the more advanced stages, structural OCT measurement of the macular ganglion cell complex^8,9,12^ and optical coherence tomographic angiography (OCTA) measurements of perfusion^11,30–34^ may perform better. The methods developed here to improve VF correlation and diagnostic accuracy could be applied to those other OCT and OCTA measurements as well.

We found that NFL_MD had significantly better glaucoma detection sensitivity at both 95% and 99% specificity diagnostic cut-points, compared to VF_MD and the best conventional NFL diagnostic parameters (overall average and inferior quadrant). While we did not intentionally optimize NFL_MD for glaucoma diagnosis, we believe the improved diagnostic performance is due to the weighted logarithmic averaging step. Converting the sector NFL measurements to a dB scale emphasizes focal defect. And weighting by VF area emphasizes the inferior and superior arcuate areas most often affected by glaucoma. To illustrate, a 5% uniform diffuse loss of NFL thickness in an average normal eye would yield an NFL_MD of −0.22 dB, well within the normal range. But a 55% loss in the inferior-most inferotemporal sector (16-division sectors), while still giving a 5% reduction in overall average NFL thickness (still within normal range), would yield an NFL_MD of −1.89 dB, which crosses the 99%-specificity diagnostic threshold for glaucoma. Glaucoma damage in the early stages tend to be focal and most likely in the sectors weighted most by VF area (inferotemporal and superotemporal). Thus the higher diagnostic accuracy NFL_MD may be due to its ability to accentuate focal loss in any of the likely sectors.

## Conclusion

In conclusion, we have developed a method to simulate VF_MD based on OCT NFL measurements. The resulting parameter is called NFL_MD. Compared to conventional NFL parameters, NFL_MD has improved correlation with VF_MD. NFL_MD is on a dB scale that corresponds to VF_MD, and thus the speed of glaucoma progression measured by NFL_MD is easier to interpret than conventional NFL parameters. NFL_MD has better reproducibility than VF_MD, thus it may allow earlier detection of significant glaucoma progression. We plan to study the use of NFL_MD in monitoring glaucoma progression using the AIG dataset in upcoming publications.

## Method

### Data

Data from the Advanced Imaging for Glaucoma (AIG) study were analyzed in this study. AIG was a bioengineering partnership (R01 EY013516) and multi-site longitudinal prospective clinical study sponsored by the National Eye Institute (ClinicalTrials.gov identifier: NCT01314326). The study design and baseline participant characteristics have been reported previously^35^, and the Manual of Procedures is publically available online (www.AIGStudy.net). The study procedures adhered to the Declaration of Helsinki, which guides studies involving human subjects. Written informed consent was obtained from all patients for the participation in the study. Proper institutional review board approvals were obtained from all participating institutions. The study was in accordance with the Health Insurance Portability and Accountability Act of 1996 (HIPAA) privacy and security regulations. This study was approved by the Institutional Review Board (IRB) of Oregon Health&Science University.

In this study, data collected from the normal (N), pre-perimetric glaucoma (PPG) and perimetric glaucoma (PG) participants from the AIG study were analyzed.

Both eyes of normal participants met the following criteria: VF tests within normal limits, IOP < 21 mm Hg, and normal optic nerve on slit-lamp biomicroscopy.

Eyes enrolled in the PPG group had glaucomatous optic neuropathy as evidenced by diffuse or localized thinning of the neuroretinal rim or NFL defect on fundus examination, but normal VF with pattern standard deviation (PSD, P > 0.05) and glaucoma hemifield test (GHT) within normal limits.

Eyes enrolled in the PG group had glaucomatous optic neuropathy as evidenced by diffuse or localized thinning of the neuroretinal rim or NFL defect on fundus examination, and corresponding repeatable VF defects with PSD (P < 0.05) or GHT outside normal limits.

Exclusion criteria common to all groups included best-corrected visual acuity (BCVA) worse than 20/40, evidence of retinal pathology, or history of keratorefractive surgery. Cataract was not an exclusion criteria for AIG enrollment, but the cataract density (grade 0 to 4) was recorded. For the analysis in this article, we excluded eyes with cataract density worse than 2 or BCVA worse than 20/30 during any of the 4 visits analyzed in this article.

Normal participants were followed every 12 months and glaucoma participants were followed every 6 months. OCT and VF testing were performed at all follow-up and baseline visits for PG/PPG participants. In order to improve the repeatability of the measurements in the same eye, we averaged measurements from the 4 earliest consecutive visits that had complete OCT and VF data for glaucoma participants.

### Visual field testing

The visual field was assessed by standard automated perimetry on the Humphrey Field Analyzer (HFA II; Carl Zeiss Meditec, Inc, Dublin, California, USA) using the Swedish Interactive Thresholding Algorithm 24-2. The minimum requirement for reliability included less than 15% fixation losses, less than 33% false positives, and less than 33% false negatives. The VF test was done at baseline for all participants, and then every 6 months for glaucoma participants and every 4 years for normal participants.

### Nerve fiber layer thickness measurement and conversion to decibel scale

#### Spectral-domain optical coherence tomography

Participants were scanned with spectral domain OCT (RTVue, Optovue, Inc, Fremont, California, USA), the optic nerve head (ONH) and 3-D Disc scans were used to map the optic nerve head and nerve fiber layer. Three ONH scans were obtained in each visit for disc and NFL thickness measurements. One Disc 3D scan was obtained at the baseline visit. The OCT data were export from the OCT machine of each clinical center and send to the OCT reading center for grading. In the OCT reading center, OCT data were analyzed using REVue software (Version 6.12, Optovue). Firstly, the center of the optic disc was identified on the Disc 3D scan, and was used to register the disc positions in all subsequent ONH scans. Then NFL thickness maps (1.3~4.9 mm) were measured from the ONH scans; a NFL thickness profile was resampled on a 3.4-mm diameter circle centered on the disc^36^. The process was automated but the grader needed to validate the data to exclude scans with poor SSI, cropping or failed segmentation. Scans with failed segmentation, cropping, low signal strength index (SSI < = 37), or decentration > 0.75 mm were excluded from further analysis. Among the repeated ONH scans in the same visit, one scan was randomly picked for further analysis and comparison to the single VF test available for each visit.

#### Sector NFL thickness

**Figure 4:**
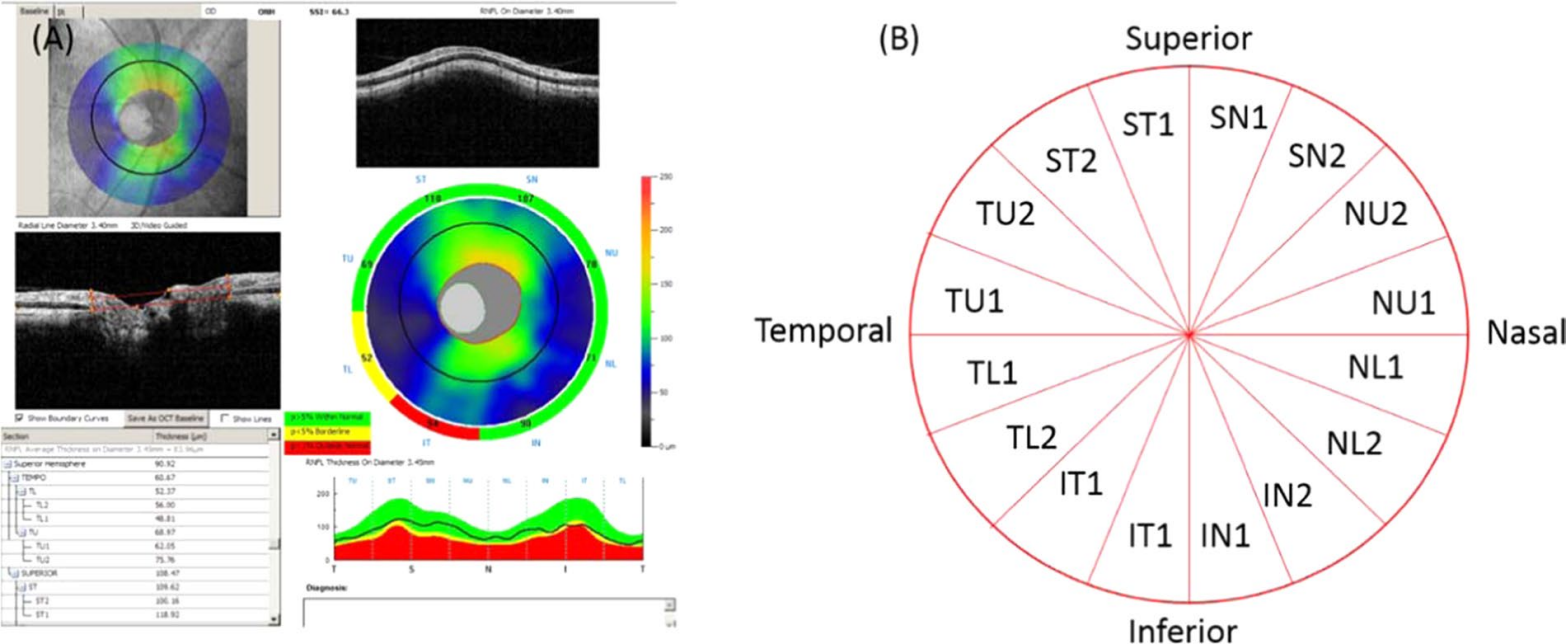
Peripapillary retinal nerve fiber layer (NFL) parameters from spectral-domain OCT. (**A**) Analysis page for the ONH scan in the RTVue Software Version 6.12. Overall, hemisphere, quadrant and sectoral average NFL thickness are included in the output parameters. (**B**) The NFL thickness was averaged in16 sectors with arc lengths of 22.5°.

The NFL thickness profile (D = 3.4 mm) was outputted as average values in 16 sectors, 4 quadrants, 2 hemispheres, and 1 overall circle (Fig. 4).

#### Age and axial length correction

In the normal group, we found significate association of NFL thickness with age, and with axial length (p < 0.001). Thus a multivariant regression was used to correct the NFL thickness. The regression is applied to each sector seperatly. Based on the regression, the sector NFL thickness was corrected to reference age and axial length. The reference age was set to 50 years to match the VF test^37^. The reference axial length was set to the average axial length (23.6 mm) of the emmetropic (spherical equivalent refraction between −1.00 and + 1.00 D) eyes in the normal group.

#### Floor value of nerve fiber layer thickness

The NFL floor value refers to the residual thickness of NFL in end stage glaucoma. This thickness represents the remaining glial tissue and secondary scar tissue. In order to estimate the fraction of nerve fibers that has been lost, it is necessary to know both the reference value from a normal population, as well as the floor value from areas of severe glaucoma damage. When 100% of the nerve fibers are present, the NFL thickness is close to the normal reference value. At the other extreme, an NFL thickness near the floor value indicates that the nerve fiber survival is near 0%. To estimate the floor value, we selected eyes with severe glaucoma according to the modified Hodapp-Parrish-Anderson criteria (VF_MD < −12 dB). In each of these eyes, the NFL sector with end-stage damage was identified as the sector with the lowest NFL thickness as a percentage of the normal reference. The residual percentage from the worst sectors of these eyes were then averaged to obtain the floor percentage. Finally, each sector’s floor value was defined as the floor percentage times the normal reference value.

#### Converting nerve fiber layer thickness to a logarithmic decibel scale

The following formula was used to transform NFL thickness on a µm scale to NFL loss on a dB scale.

$$NF{L}_{dB}=10\times \,\log \,10(\frac{NF{L}_{{\rm{\mu }}m}-{\rm{f}}}{{\rm{N}}-{\rm{f}}})$$

where f was the floor; N was the normal reference (average value of healthy eyes in our normal group). This conversion formula could be applied to either overall or sector NFL thickness values.

The normal reference and floor were adjusted for age and axial length in the above formula. Multiple linear regression was performed to fit axial length and age to NFL thickness for each sectoral, quadrantile or overall average. Then the normal references were generated from the fitting equation. The floor value for NFL thickness was adjusted for axial length, but not age^9^.

We limited the minimum value of NFL_dB_ to −12.8 dB to avoid extremely negative dB values that could be obtained when NFL thickness is near the floor. The −12.8 dB minimum is equivalent to 5% above the floor value. This limit was based on the coefficient of variation of sector NFL thickness of 5% for repeat measurements in normal eyes.

### Weighted logarithm average of sector NFL thickness

**Figure 5:**
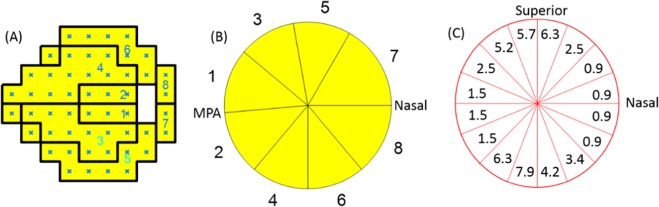
Weighting of nerve fiber layer (NFL) sectors used to calculate NFL mean deviation (MD). (**A**) The modified Garway-Heath visual field (VF) sectors. (**B**) The circumpapillary NFL thickness profile is divided into 8 sectors that correspond to the 8 VF sectors. The weights in these sectors correspond to the number of VF test points. (**C**) The weights (numbers shown in the pie slices) for 16 evenly divided NFL sectors was obtained by interpolation of the 8 sectors in B. Abbreviation: MPA - maculopapillary axis.

In order to simulate the VF_MD, we calculated a weighted average of sector NFL_dB_. The weight was set to the VF area corresponding to NFL bundles passing through a particular peripapillary sector. To determine weights, we used a modified Garway-Heath scheme to estimate the VF area (Fig. 5). The 6 sectors of the original Garway-Heath scheme were divided into 8 sectors by adding superior-inferior divisions^25,38^. In the VF map, the test points were divided along the horizontal center line. In the peripapillary profile, the dividing line was the maculopapillary axis temporally and the horizontal midline nasally. The Garway-Heath sectors were originally defined at the disc rim; we extended these sector divisions outward from the disc edge to the 3.4-mm diameter circle D = 3.4 mm along the average trajectory of nerve fibers obtained using a published flux analysis in normal human subjects^39^. The weight in the 8 sectors was set to the number of VF test points in corresponding VF sector. These weights in these 8 sectors were interpolated to obtain weights for the 16 evenly divided sectors (Fig. 2C). With these weights, we calculated the NFL weighted logarithm average (NFL_WLA_) using the following formula:

$$NF{L}_{WLA}=\frac{1}{52}\mathop{\sum }\limits_{i=1}^{16}{w}_{i}\times NF{L}_{dB}(i)$$

Where w_i_ is the weight of a sector i; NFL_dB_(i) is the NFL loss in dB for sector I; the number 52 is the summation of weights.

### Simulation of visual field mean deviation

In order to reduce measurement noise, we averaged NFL parameters and VF_MD from 4 consecutive visits for glaucoma eyes. The first 4 consecutive visits with Spectral domain OCT scans were selected. When VF_MD was plotted against NFL_WLA_, it was clear that the relationship was still significantly nonlinear. Thus a quadratic regression was used to fit the NFL_WLA_ to VF_MD using all eyes from normal, PPG and PG groups. The intercept was fixed at zero with the *a priori* knowledge that an average normal NFL thickness profile should correspond to an average normal VF. Five-fold cross validation was used to avoid bias due to overfitting. For each fold, NFL-MD was then estimated in the validation sub-set using the corresponding fitting result. The NFL-MD obtained in 5 folds were pooled for the statistic analysis.

### Statistical analysis

To remove the between-eye correlation, the linear mixed effects model was used to compare the mean values of parameters between groups. Chi-square test was used for comparing gender between groups. Linear mixel effects model was applied to estimate the pearson correlation and the spearman correlation coeffcients between NFL parameters and VF MD^40,41^. A percentile bootstrap method was used to compare the correlation coefficients^42^.

To assess the between-visit reproducibility, the residual of linear regression over time was calculated for the 4 consecutive visits in glaucoma eyes. This was applied to the overall NFL thickness, NFL_MD, and VF_MD. The residuals were pooled by groups stratified by glaucoma severity. Glaucoma severity was staged by a modified Hodapp-Parrish-Anderson (HPA) classification system: Stage 0 - PPG, Stage 1 - early PG (MD > = −6 dB), Stage 2 moderate PG (−12 dB < = MD < −6 dB), and Stage 3 - severe PG (MD < −12 dB)^7^.

Intra-class correlation was used to compare the within-visit repeatability and the between-visit reproducibility of the overall NFL thickness average and NFL_MD^43^. The within-visit repeatability was based on scans in baseline visits. The between-visit reproducibility was based on pairwise analysis between the baseline and the first follow-up visit.

To assess agreement, the difference between NFL_MD and VF_MD was calculated in each eye from each visit. The mean difference was averaged over the 4 consecutive visits and then averaged again in each of the 4 stages. The standard deviation was calculated by pooling the difference over the 4 consecutive visits by root mean square. Then it is pooled again in each of the 4 stages. Difference between NFL_MD and VF_MD was also assessed by Bland-Altman analysis. Agreement between NFL_MD and VF_MD for glaucoma staging was assessed by the F1-score.

The diagnostic accuracy of separating PPG and PG groups from the normal group were evaluated by the area under the receiver operating characteristic curves (AROC), and the sensitivities at 95% and 99% specificity cutoffs with Generalized estimating equations^43,44^. The cutoff thresholds were based on the mean and standard deviation from normal eyes after the age and axial length adjustment, assuming normal distribution. The 95%/99% specificity cutoff was set at 1.65/2.33 standard deviations (SD) below the mean of the normal group. The overall and inferior NFL thickness values had a normal distribution in the normal group according to the Kolmogorov-Smirnov normality test. VF_MD and NFL_MD had normal distributions only after transformation from dB to linear scale, therefore their diagnostic cutoff values were calculated on the linear scale and then transformed back to the dB scale.

All statistical analyses were done using MATLAB with the statistical toolbox.
